# Sirtuins as Mediator of the Anti-Ageing Effects of Calorie Restriction in Skeletal and Cardiac Muscle

**DOI:** 10.3390/ijms19040928

**Published:** 2018-03-21

**Authors:** Alberto Zullo, Emanuela Simone, Maddalena Grimaldi, Vincenzina Musto, Francesco Paolo Mancini

**Affiliations:** 1 Department of Sciences and Technologies, University of Sannio, 82100 Benevento, Italy; emanuela.simone1@gmail.com (E.S.); vincenzina.musto@icloud.com (V.M.); 2 CEINGE Biotecnologie Avanzate s.c.ar.l., 80145 Naples, Italy; 3 Department of Pediatric Oncology and Hematology, Charité University Hospital, 13353 Berlin, Germany; grimaldi.madda@gmail.com

**Keywords:** sirtuins, calorie restriction, ageing, nutrient deprivation, skeletal muscle, cardiac muscle

## Abstract

Fighting diseases and controlling the signs of ageing are the major goals of biomedicine. Sirtuins, enzymes with mainly deacetylating activity, could be pivotal targets of novel preventive and therapeutic strategies to reach such aims. Scientific proofs are accumulating in experimental models, but, to a minor extent, also in humans, that the ancient practice of calorie restriction could prove an effective way to prevent several degenerative diseases and to postpone the detrimental signs of ageing. In the present review, we summarize the evidence about the central role of sirtuins in mediating the beneficial effects of calorie restriction in skeletal and cardiac muscle since these tissues are greatly damaged by diseases and advancing years. Moreover, we entertain the possibility that the identification of sirtuin activators that mimic calorie restriction could provide the benefits without the inconvenience of this dietary style.

## 1. Introduction

Environmental factors profoundly affect the fate of living organisms and nutrition is one of the most influential factors. Nowadays longevity is a major goal of medical science and has always been a chimera for the human being since ancient times. In particular, efforts are aimed at achieving successful ageing, namely a long life in the absence of serious diseases, with a good level of physical and mental independence and adequate social relationships [1].

Accumulating data clearly demonstrates that it is possible to influence the signs of ageing. Indeed, nutritional interventions can promote health and longevity. A tribute must be given to Ancel Keys, who was the first one to provide solid scientific evidence about the role of nutrition in the health/disease balance at the population level, specifically in relation to cardiovascular disease, still the leading cause of death worldwide [2,3,4]. It is generally appreciated that the type of diet can profoundly influence the quality and quantity of life and the Mediterranean diet is paradigmatic of a beneficial dietary pattern [5,6,7].

The growing consciousness of the beneficial effects of a specific dietary pattern on health and longevity in the second half of the last century generated a powerful thrust toward designing diets that could reduce the risk of chronic diseases, thus resulting in healthy ageing. Thus, in the 1990s the Dietary Approaches to Stop Hypertension (DASH) diet was devised in order to evaluate whether it was possible to treat hypertension non pharmacologically [8]. Indeed, the DASH diet was quite similar to the Mediterranean Diet, being rich in fruit and vegetable, whole grains, and fibers, while poor in animal saturated fats and cholesterol. The very good news coming out of the study was that not only did the DASH diet lower blood pressure, but it also reduced the risk of cardiovascular disease, type 2 diabetes, some types of cancer, and other aging-associated diseases [9,10]. To further improve the health benefits of plant food-rich, animal fat-poor diets, particularly in hypercholesterolemic individuals, the Portfolio Diet was designed [11]. This diet, besides being largely vegetarian, with only small amounts of saturated fats, recommends also a high intake of functional foods, including viscous fibers, plant stanols, soy proteins, and almonds. Interestingly, participants on the Portfolio Diet exhibited a reduction of coronary heart disease risk associated with lower plasma cholesterol and inflammatory indexes in comparison to participants on a healthy, mainly vegetarian diet [12].

However, also the amount of ingested food has been attracting the interest of the scientific community as a potential modifier of the balance between health and disease in many different living species. In particular, calorie restriction (CR) has been demonstrated to be an emerging nutritional intervention that stimulates the anti-ageing mechanisms in the body [13,14,15,16,17,18,19,20,21,22]. Therefore, the diet of the people living on the Japanese island of Okinawa has been extensively analyzed because these islanders are well-known for their longevity and increased health span, resulting in the greatest frequency of centenarians in the world [23]. Very interestingly, the traditional Okinawan diet resulted to be very similar to the Mediterranean Diet and the DASH diet in terms of food types [23]. However, the energy intake of Okinawans, at the time of the initial scientific observations, was about 20% lower than the average energy intake of the Japanese, thus determining a typical condition of CR [24].

CR causes life extension through mechanisms that involve different molecular players among which sirtuins, a family of nutrient-sensing proteins, take center stage. Sirtuins have different, NAD^+^-dependent enzymatic activities, among which the deacylating one is common to all the isoforms [25]. Sirtuins are expressed in several tissues, including skeletal muscle, which is particularly relevant in ageing, due to its influence on metabolism and its gradual loss as organisms get older. In this review, we focus on the role of sirtuins in mediating the anti-ageing effects of CR in skeletal and cardiac muscle (Table 1).

## 2. Calorie Restriction, Sirtuins, and Ageing

CR is a dietary regimen with a 20–40% reduction of the normal daily calorie intake without malnutrition [26]. The effect of CR on ageing was first shown by McCay and colleagues in an animal model over 80 years ago [27]. They observed that calorie-resticted mice lived longer compared to control mice on a regular diet. These early results have been confirmed later by many other studies in different species, from unicellular yeast to non-human primates [15,16,17,18,19,20,21,22,28,29,30,31].

However, while two of the three available studies demonstrated increased survival in calorie-restricted rhesus monkeys, the third study did not report any significant effect of CR on the survival of the experimental animals [15,32,33]. Interestingly, the same authors of two out of the three contrasting papers reassessed their studies together. As results, they identified the differences in experimental design that could explain, at least in part, the diverging outcomes and concluded that CR was indeed able to improve both health and survival in monkeys [34]. At this point, it is conceivable to speculate that the same mechanisms and effects on health and survival could also take place in humans practicing CR. Yet, there are no long-term human studies to validate this hypothesis. Indirect evidence comes from historical episodes, such as the forced reduction of food intake in Danish people during the World War 1 that led to a reduction of 34% of death rates, and, similarly, a forced 20% CR in the population of Oslo, during World War 2 that brought about a 30% reduction of mortality [35,36]. A natural experiment is provided by the already mentioned population of the Japanese island of Okinawa, where 4–5 times higher incidence of centenarians compared to any other industrialized country was registered, in association with a spontaneous calorie-restricted diet due to the frugal nutritional habits of the Okinawans [37].

As firstly demonstrated in animal models, CR delays the detrimental consequences of the signs of ageing, and this is a key point underlying the health effects of CR. In particular, CR is inversely correlated with the onset of age-related diseases, such as diabetes, atherosclerosis, cardiomyopathy, kidney disease, sarcopenia, respiratory disease and cancer [14,38]. In rodents and monkeys, CR reduces insulin resistance, glucose intolerance, cognitive decline and immune dysfunction [34,39,40,41]. Moreover, it has been demonstrated that CR also attenuates the age-related neurodegeneration, sarcopenia, and auditory loss [42]. Importantly, these metabolic and functional effects of CR are long-lasting [34].

So far, some observational and randomized clinical trials have been performed also in humans. They confirmed the anti-ageing effects of CR, including a delayed onset of the most common chronic diseases, such as type 2 diabetes, cardiovascular diseases, and cancer. In particular, the metabolic and molecular changes induced by CR in humans are similar to those promoting health and prolonging life of CR-treated animals [42].

**Figure 1:**
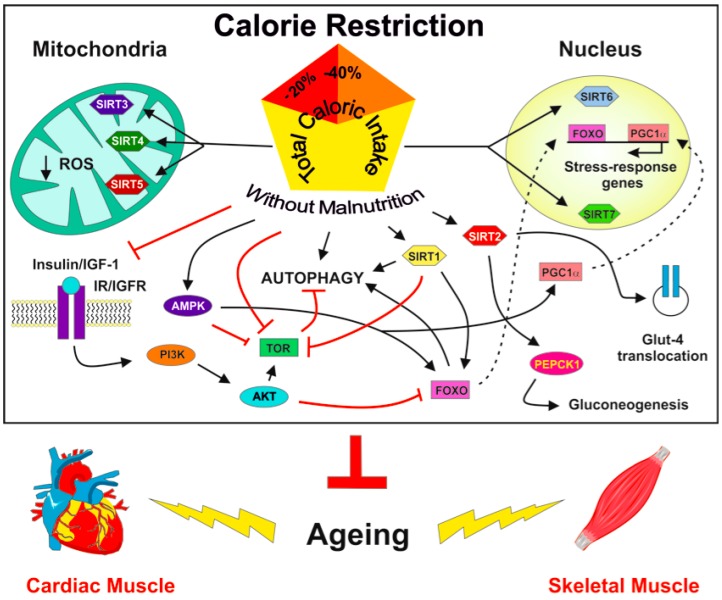
Molecular mechanisms underlying the sirtuin-mediated anti-ageing effects of Calorie Restriction in cardiac and skeletal muscle. CR can modulate sirtuin activity in different cell compartments, either cytoplasm, or mitochondria, or nucleus. Abbreviations: CR, calorie restriction; PI3K, phosphatidylinositol-3-kinase; AKT, protein kinase B; TOR, target of rapamycin; AMPK, AMP-activated protein kinase; FOXO, forkhead box O transcription factor; GLUT-4, glucose transporter type 4; PEPCK1, phosphoenolpyruvate carboxykinase 1; PGC1α, proliferator-activated receptor-gamma coactivator-1α; IR, insulin receptor; IGF1, insulin-like growth factor1; IGFR, insulin-like growth factor receptor.

At a molecular level (Figure 1), the effects of CR involve nutrient-signaling pathways, such as those of the mammalian target of rapamycin (mTOR), the insulin-like growth factor 1 (IGF-1), and a family of seven proteins named sirtuins [31]. Sirtuins target proteins involved in different processes, such as DNA repair, DNA stability, epigenetic modification of chromatin, stress resistance, cell cycle progression, reactive oxygen species (ROS) production and metabolism, mitochondrial function and autophagy [43]. Several pathways can activate sirtuins following CR. For instance, the upregulation of endothelial nitric oxide synthase (eNOS), which is a very important effector of CR, can activate sirtuin 1 (SIRT1) [44,45,46]. Then, sirtuin activation modifies the acetylation/deacetylation balance, a crucial mechanism for mediating the metabolic responses to changes in nutrient availability [47]. Many of the proteins targeted by acetylation are localized in the mitochondria and are involved in the tricarboxylic acid cycle, β-oxidation, and oxidative phosphorylation [47]. Nutritional stimuli not only influence mitochondrial biochemical pathways, but also affect the number and life cycle of these organelles. Indeed, CR, via inhibition of IGF-1 and mTOR pathways and activation of AMP-activated protein kinase (AMPK) and sirtuins, modulates oxidative metabolism as well as the biogenesis and turnover of mitochondria. AMPK and peroxisome proliferator-activated receptor gamma coactivator 1-alpha (PGC-1α) are key players in these pathways. CR also lowers ROS production through enhanced mitochondrial aerobic metabolism and increased activity of antioxidant enzymes [48]. 

Aerobic metabolism is the most efficient energy-producing process in living organisms, thus, CR improves metabolic efficiency, an effect that fits very well with the CR-associated lower metabolic rate. Moreover, CR enhances fatty acid oxidation, while reducing carbohydrate oxidation [49,50]. 

Beside regulating cellular activities at the cytoplasmic and organelle levels, sirtuins also mediate the response to CR at the nuclear level, although some issues remain still open, such as the relationship between CR and telomere length. Telomere shortening is an important biomarker of cell senescence and CR has been demonstrated to decrease the rate of telomere shortening in various mouse tissues during ageing [44]. In particular, CR enhances the activity of telomerase, probably through the regulation of telomere-associated proteins [51,52,53]. On the contrary, the effects of CR on telomere length has been questioned by other studies in humans and rhesus monkeys [54,55]. DNA integrity is another target of the nuclear activity of sirtuins, following CR. DNA integrity is fundamental for the health of cells and organisms and is guaranteed by the protection from DNA damaging agents and the activity of DNA repair systems [56]. CR both reduces DNA damage from oxidative stress and stimulates DNA repair by supporting the expression of genes involved in the anti-oxidant defense and in the nucleotide excision repair process [57,58,59,60]. Therefore, experimental evidence so far collected supports the hypothesis that CR hinders the age-related accumulation of DNA damage and reduction in DNA repair capacity.

The effects of ageing are exacerbated also by the progressive reduction of stem cell activity and, thus, by the reduced regeneration potential of tissues. Tissue regeneration is a delicate and complex process allowing the maintenance of body integrity, wellness and suitable lifespan [61,62,63,64,65]. In this regard, it has been shown that CR promotes also the activity of stem cells, thus preserving their regenerative potential. Studies on mice suggest that CR sustains the health and survival of stem cells, allowing the maintenance of adequate stem cell pools in different tissues [34,66,67,68]. Indeed, CR reduces hematopoietic stem cell (HSC) ageing and increases HSC quiescence, thus enhancing the repopulation capacity of HSCs [69]. Moreover, CR augments the number of neural stem cells in the brain of adult female animals [70]. CR also promotes self-renewal of intestinal stem cells, improves their function and their niche. These effects lead to an enhanced regeneration potential of the intestine [71]. In skeletal muscle, CR improves satellite stem cell availability and activity, thus promoting a more effective recovery from injury [72]. The molecular mechanisms involved in the stem cell response to CR include mTOR, AMPK/LKB1, FOXOs, sirtuins, IGF-I, PI3K/AKT; PTEN and the authophagic machinery [68,73].

The comprehension of the molecular pathways promoting both health span and lifespan, has led to the identification of natural and synthetic compounds with anti-ageing effects. This is the case of resveratrol, a polyphenol from grapes and the anti-diabetic drug metformin. These molecules have been demonstrated to be sirtuin activators and to hinder the deleterious effects of ageing by modulating some of the same molecular pathways activated by CR [74,75,76,77,78,79,80,81,82,83,84].

## 3. CR and Sirtuins in Skeletal Muscle

In mammals, skeletal muscle undergoes a progressive age-dependent loss of mass, function and regenerative potential [85]. This process contributes to a continued metabolic derangement, a decay in the quality of life and a consequent reduction of health- and lifespan. The function of skeletal muscle in the whole body extends far beyond the locomotion. In fact, this tissue acts also as an amino acid stock for protein synthesis, contributes to glucose uptake from the bloodstream, and secretes cytokines and growth factors (myokines) into the bloodstream, thus regulating the activity of other tissues [85]. The progressive loss of strength, which is typical of the ageing muscle, is also related to the compromised function and reduced number of satellite, stem, and progenitor cells. This also leads to a reduced muscle repair and regeneration potential in aged organisms [48,68]. Indeed, skeletal muscle is very susceptible to changes in nutrient availability and in vitro, the availability of glucose and amino acid strongly influences the myogenic potential of skeletal muscle satellite cells [86,87]. CR delays the age-dependent reduction in skeletal muscle mass, as demonstrated in animal models, and this effect is mediated by the protection of DNA and mitochondria from damage, stimulation of autophagy, and inhibition of inflammation [88,89,90,91]. During CR, cells undergo a transcriptional shift from the activation of genes promoting growth to those supporting maintenance/repair [92]. In this regard, the analysis of gene expression in skeletal muscle of aged mice under CR revealed an upregulation of genes involved protein turnover with a concomitant lower level of macromolecular damages [93].

Interestingly, some muscular effects of CR are also depending on age, sex and genetics [82,93,94,95]. In particular, dietary condition has been seen to influence ageing-related changes in gene expression of mouse skeletal muscle only if it occurs at the juvenile, but not the adult stage [82,93,94]. Furthermore, CR counteracts the age-dependent increase in systemic chronic inflammation and insulin resistance by modulating the glutathione redox status, nuclear factor kappa-light-chain-enhancer of activated B cells (NF-κB), SIRT1, peroxisome proliferator-activated receptors (PPARs), and forkhead box O (FOXOs) [96,97]. This is particularly relevant because age-related chronic inflammation negatively influences the regenerative potential of skeletal muscle and skeletal muscle mass [48,98]. Beside reduced inflammation, CR-treated mice had increased plasma cortisol level, and enhanced levels of chaperones and autophagy complexes involved in protein folding and turnover [47]. Although elevated cortisol is associated with adverse effects, including increased protein catabolism, this hormone is also a powerful anti-inflammatory agent. Moreover, humans do not respond to CR with a biologically relevant increase of serum cortisol [99]. In skeletal muscle, ageing is also associated with increased DNA damage, inhibition of mitochondrial function and reduced energy metabolism and muscle performances. In particular, the increased activity of the DNA-dependent protein kinase in aged organisms is responsible for the metabolic dysregulation and the decay of physical performances [100].

Intriguingly, CR can synergize with physical exercise in reducing the negative effects of ageing on muscular mass and efficiency. Physical workout is the major stimulus for muscle trophism and must go together with adequate supply of nutrients, particularly essential aminoacids and vitamins in order to maintain or increase muscular efficiency. Therefore, it is important to recall the significance of CR in the present context, which is a reduction of calorie intake in the absolute absence of any deficiency of essential nutrients. Having said that, CR, together with physical exercise, can be effective in hindering the age-related changes in sarcolemmal proteins, and therefore, contrasting the decline in muscle performances of rats. In particular, this observation refers to the membrane repair proteins MG53, dysferlin, annexin A6, and annexin A2 and Nox2 subunits [101]. However, the effects of CR and exercise on muscle are different. Indeed, CR was able to increase insulin-stimulated glucose uptake in the soleus muscle of old rats, whereas physical exercise did not [102]. A similar effect of CR in rat skeletal muscle was observed in another study, without changes in the content of mitochondrial hexokinase II, or electron transport chain and oxidative phosphorylation proteins [103].

There are several other pathways that can be influenced by CR in the muscle and that can positively affect this tissue. A major one recognizes as central player mTOR, a kinase that regulates skeletal muscle growth, by phosphorylating P70S6 kinase 1 (S6K1) [104,105,106]. It is also worth mentioning IGF-1, since in aged skeletal muscle circulating IGF-1 and its downstream intracellular signaling are reduced. In particular, there is a reduction in the activity of phosphoinositide 3-kinase, AKT, mTOR, p70S6K1, 4E-BP1 and eukaryotic translation initiation factor 2B [55,107,108,109,110]. Interestingly, this reduction of IGF-1 activity is associated with both reduction of skeletal muscle mass and with increased life- and health-spans [111].

The tapering muscular function, which characterizes ageing and chronic diseases, is influenced by nutrient availability, redox state, and the ratio of NAD^+^ to NADH. Therefore, SIRT1 plays a crucial role in the balance between healthy and defective skeletal muscle. Indeed, in vitro studies demonstrated that SIRT1 expression prompts an anti-oxidative response in skeletal muscle cells after mechanical stress [112]. Moreover, SIRT1 and AMPK are crucial mediators of the effects of CR in mouse skeletal muscle [113].

Exposure of cultured myoblasts to glucose restriction blocks their differentiation into myotubes and increases the activity of SIRT1 by AMPK-dependent regulation of nicotinamide phosphoribosyltransferase (NAMPT), the enzyme responsible for NAD^+^ turnover [114,115]. In myotubes cultured in the presence of low glucose concentration, SIRT1 is necessary for the metabolic switch from glucose utilization to fatty acid oxidation; this important response is present during CR and is reduced during metabolic diseases [116].

SIRT1 produces these and other effects by acting on/with its substrates and transcriptional/epigenetic co-factors, including PGC-1α, hepatocyte nuclear factor 4 alpha, p53, FOXOs, sterol regulatory element-binding protein 1 (SREBP-1C) PPARγ, NF-κB, Ku70, P300/CBP-associated factor, myogenic differentiation factor (MYOD), myocyte enhancer factor-2 (MEF2), signal transducer and activator of transcription 3, heat shock transcription factor 1, Smad7, suppressor of variegation 3-9 homolog 1, enhancer of zeste 2 polycomb repressive complex 2 subunit, nucleomethylin, eNOSC and various histones [117,118,119,120]. SIRT1 deacetylates SREBP-1C, thus enhancing its activity and the expression of its target genes in mouse skeletal muscle [121]. Moreover, SIRT1 participates in the response to injury in muscle, by modulating both NF-κB and FOXOs and regulating MYOD and MEF2. In fact, NF-κB and FOXOs regulate the muscle-specific RING finger protein 1 and the muscle atrophy F-box protein/atrogin-1, which are E3 ubiquitin ligases involved in proteasome-mediated proteolysis of muscle proteins [122].

Gene expression in skeletal and heart muscle is regulated in a circadian fashion by the CLOCK:BMAL1 transcription factor, that controls the expression of other clock genes. This endogenous rhythm can be influenced by external stimuli, such as light and food availability that act on the hypothalamic suprachiasmatic nucleus [123]. SIRT1 deacetylates CLOCK and BMAL1 in a circadian fashion in mouse embryonic fibroblasts and is an important component of the circadian clock. NAD^+^ plays a central role in both metabolism and circadian rhythm and controls the NAMPT production through SIRT1-mediated CLOCK and BMAL1 activation. In the same way, the circadian clock controls intracellular NAD^+^ levels by a transcriptional feedback loop [123]. Moreover, SIRT1 is also able to promote proliferation of muscle cell precursors by blocking the expression of cell cycle inhibitors and hindering their transformation into fully differentiated myocytes [111,124,125]. This observation has been made in cultured murine myotubes and has been associated with SIRT1-mediated repression of MYOD, a major muscle transcriptional regulator, and of MEF2 [114,126]. In turn, repression of MYOD downregulates the expression of myogenin and several contractile proteins [114].

SIRT1 could be important for the regeneration of skeletal muscle and heart, by inducing proliferation and differentiation of their respective adult stem cells. Moreover, the activity of SIRT1 on FOXO transcription factors regulates the catabolic pathways of proteins, blocks apoptosis and promotes oxidative stress resistance and DNA repair [127]. In chronic inflammation or physical inactivity, SIRT1 sustains skeletal muscle survival, thus counteracting muscular atrophy [111]. Most importantly, CR (particularly glucose restriction), but also physical exercise enhance SIRT1 expression through AMPK, FOXO3A and EGR1 activities, that, in turn, leads to activation of FOXO4 and PGC-1α. These factors induce the oxidative metabolism and an anti-oxidative response in muscle cells [126].

SIRT1 and 6 are important downregulators of IGF-1 and its target signaling complex AKT/mTOR [128]. This mechanism engages SIRT6 into the homeostasis of the muscle since it has been demonstrated that the alteration of the AKT-mTOR pathway is involved in the evolution of sarcopenia [129]. In addition, SIRT6, together with SIRT1 inhibits NF-κB, thus attenuating its inflammatory signaling cascade and contrasting DNA damage, cellular senescence and oxidative stress [130,131,132].

SIRT2 has been demonstrated to be responsible for the reduction of glucose transporter 4 translocation to the sarcolemma in response to insulin in transgenic mice [133]. The expression of SIRT2 is negatively correlated with insulin sensitivity, as demonstrated in vitro in C_2_C_12_ skeletal muscle cells [134]. Interestingly, SIRT2 is also related to CR since it regulates metabolism during fasting by deacetylating phosphoenolpyruvate carboxykinase [135].

CR upregulates SIRT3 in skeletal muscle [136]. This sirtuin is localized within the mitochondria where it modulates the activity of several proteins, such as succinate-dehydrogenase, manganese-dependent superoxide dismutase (Mn-SOD) and FOXO3. The absence of SIRT3 leads to a downregulation of PGC-1α [136]. SIRT3 is also involved in the regulation of acetate metabolism that is relevant during ageing and fasting or starvation [137,138,139]. In particular, the enzymes acetyl-CoA synthase type 1 and 2 are activated by SIRT3 [137,138,139]. This activation is associated with increased lifespan in yeast [140]. During fasting, in skeletal muscle, the expression of SIRT3 is increased and the SIRT1-dependent deacetylation of PGC-1α is increased, thus, enhancing mitochondrial oxidative phosphorylation [116,136,141].

The evidence relating SIRT4 and 7 to muscle pathophysiology and CR is limited. It has been demonstrated in mouse skeletal muscle that AMPK regulates metabolism by modulating SIRT4 and 1 activity [142]. Another study analyzed the expression of SIRT7 in young and old rats and observed tissue-dependent changes in gene expression occurring during ageing and during periods of CR [143].

Interestingly, although several studies demonstrated a positive effect of CR on skeletal muscle, a recent study on mice provided contrasting data, thus, suggesting caution in considering the advantages of CR on skeletal muscle health. Moreover, long-term, CR reduces the amount of protein in rat skeletal muscle through reduction of the mTORC1 pathway [144].

## 4. CR and Sirtuins in Cardiac Muscle

In the last decades, evidence has been provided about the beneficial effects of CR on the cardiovascular system. These effects, as in other tissues, are mediated by the activation of the sirtuin genes [48] However, the expression and activity of several sirtuins is decreased, and protein acetylation increased, in the presence of cardiovascular diseases [145]. Sirtuins can affect cardiac and endothelial cells either directly or indirectly by systemic regulation. Indeed, SIRT1 has been demonstrated to be protective against endothelial dysfunction, atherothrombosis, and myocardial infarction [146]. Interestingly, the interplay between sirtuins and beta-adrenergic receptors has been hypothesized, thus opening a new scenario for the molecular changes occurring in the heart during ageing and diseases [147].

SIRT1, 2, 3 and 7 regulate cell survival in stressing or unfavorable conditions, such as ischemia/reperfusion (I/R) injury, ageing, and increase of oxidative stress [148]. SIRT3 and 6 hinder cardiac hypertrophy and heart failure [148]. Short-term CR in rats increases the expression of SIRT1–4 and 7 [149].

CR activates the autophagic machinery, which is important for the survival and the resistance of cardiomyocytes to stress. However, the efficacy of SIRT1-induced autophagy progressively deteriorates with ageing leading to a progressive decline of cardiomyocyte health and ischemic resistance, and, consequently, to heart dysfunction and failure [150,151]. This important mechanism is regulated by the SIRT1/FOXO1 pathway following nutrient deprivation.

In general, oxidative stress is a very important factor in the onset of cardiovascular disease as a consequence of its detrimental effects on cardiac and vascular cells, and of the oxidative modifications of lipoproteins that potently accelerate atherosclerosis [152]. Therefore, it is worth mentioning the well-documented contribution of CR to cellular anti-oxidative activity. Moreover, CR exerts beneficial effects on the cardiovascular system by reducing inflammation, ameliorating the insulin sensitivity and preventing cardiomyocyte apoptotic death [152]. The connection among CR, reduced inflammation, and heart benefits is reinforced by stimulation of ghrelin signaling, which has a powerful anti-inflammatory activity and leads to increased SIRT1 protein expression in mouse heart, thus, ameliorating the conditions of aged hearts. Therefore, it has been proposed that CR exerts its beneficial effects also through ghrelin signaling [153].

CR may protect heart function by also controlling adverse rises of blood pressure and by empowering the resilience of cardiomyocytes to pro-apoptotic conditions. It has been reported that CR has antihypertensive and cardioprotective effects against I/R injury in mice and rats. These effects are mediated by the activity of nitric oxide which, in turn, activates SIRT1 and its pathway [152,154,155]. In particular, the protective effect against I/R injury depends on the activation of the cardiac complement component 3 [154].

The beneficial effects of CR against the deleterious effects of ageing are mediated by inhibition of mTOR signaling, and enhancement of the activity of sirtuins and KLOTHO [156]. It has been suggested that the interplay among these three pathways could be relevant in the contest of anti-ageing approaches [156]. There is a definite time window for the healthy effects of CR. Indeed, it has been recently demonstrated that CR is beneficial only when initiated in middle or old age in mice [157]. In mice under CR, FOXO1 mediates anti-oxidant, autophagic, and anti-apoptotic responses by cardiac cells [52,53]. More interestingly, FOXO1 has been suggested as a positive regulator of telomerase activity without telomere shortening [52,53]. The length of telomeres is a marker of cell senescence, and its attrition is associated with a decay in the function and the proliferative potential of cells. This phenomenon also occurs in cardiac cells, thus resulting in the development of heart diseases [53,158,159]. CR is able to modulate the activity of telomerase and to reduce the shortening of telomeres [53,158,159].

SIRT1 expression is sensitive to environmental changes and to stress, but its response varies according to different triggering factors [148]. Moreover, SIRT1 activity is also strongly dependent on post-translational modifications [160,161]. Interestingly, the role of SIRT1 in mouse heart is strictly dose-dependent. Indeed, moderate cardiac SIRT1 expression is associated with protection from senescence, hypertrophy, apoptosis, fibrosis, heart dysfunction and oxidative stress; on the contrary, high SIRT1 expression leads to an increase of oxidative stress, apoptosis and hypertrophy and cardiac dysfunction [162].

It has been demonstrated (in vivo) that the expression and the activity of SIRT1 must be strictly controlled to end up with a specific cardiomyocyte response [163]. In fact, 2.5–7.5 cardiac overexpression of SIRT1 is beneficial for the heart, hindering the age-dependent hypertrophy and reducing the detrimental effects of I/R injury. The molecular mechanism underlying this effect is the activation of protective genes, such as Mn-SOD, thioredoxin 1, and BCL2-like protein and the downregulation of proapoptotic genes such as BCL2-associated X protein [164]. 

On the contrary, a marked overexpression of SIRT1 (12.5 times) in heart is associated with mitochondrial dysfunction, low ATP content, decreased citrate synthase activity, increased oxidative stress, and significantly reduced PGC-1α expression, thus leading to cardiac dysfunction [162]. Thus, in heart, a controlled SIRT1 expression could attenuate the detrimental effects of ageing, although the total ablation of SIRT1 expression, as realized in SIRT1 knockout (KO) mice, could reduce the negative heart remodeling in response to hypertrophic stimuli [165]. The molecular mechanisms underlying the effects of SIRT1 on cardiac hypertrophic response to pressure overload implicate various molecules and pathways, including PPARα, estrogen-related receptors, AKT and pyruvate dehydrogenase kinase 1 (PDK1) [165,166].

Besides SIRT1, SIRT2, 3, 6 and 7 have been involved in heart pathophysiology. Although the role of SIRT2 in the heart is not well characterized, some reports indicate that this protein is involved in the development of the heart and suggest that it may be also involved in the development of cardiac hypertrophy by activating AKT signaling [163].

SIRT3 has been demonstrated to be a key factor in the anti-oxidant effect of CR [146]. This protein exerts its deacetylase activity on mitochondrial proteins and regulates the turnover of mitochondria. Experiments on SIRT3 KO mice demonstrated that this protein is involved in the regulation of proteins of the ATP production machinery and in the age-dependent development of hypertrophy in the heart [163]. In this context, SIRT3 promotes resistance to the onset of hypertrophy, cardiomyopathy, oxidative stress damage, and metabolic dysregulation [146].

The anti-hypertrophic activity of SIRT3 is mediated by the Mn-SOD and catalase genes for their effect in diminishing the oxidative stress; by cyclophilin D, which triggers the mitochondrial permeability transition pore opening, and thus cell death; by liver kinase B1-dependent activation of AMPK [167,168,169]. Moreover, the anti-ageing activity of SIRT3 relies also on the glycogen synthase kinase 3 beta-mediated block of transforming growth factor beta 1 signaling and its fibrotic activity [170]. Collectively, all data seem to indicate that SIRT3 activity has beneficial effects on the heart by improving mitochondrial activity and health.

Differently from other sirtuins, the evidence relating SIRT4 and 5 with heart pathophysiology is scarce, mainly relies on in vitro studies, and suggests a protective role of these sirtuins in cardiac cells [171,172,173]. These sirtuins regulate mitochondrial metabolism and are involved in cellular energy and redox balance in cardiac cells [174].

Concerning SIRT6, it hinders dyslipidemia, cell senescence, and cardiac hypertrophy [146,175]. A very recent evidence indicates that SIRT6 regulates the transcription of PDK4 via FOXO1. This, in turn, has a detrimental effect on mitochondrial activity [176].

As shown in SIRT7 KO mice, this protein is implicated in the cardiac protection from hypertrophy and inflammation-mediated fibrosis [177]. The mechanism is based on the activation of AKT pathway [177]. Moreover, SIRT7 is involved in regulating lipid metabolism and contrasting the onset of cardiac diseases [146]. 

Apart from the experimental evidence supporting a positive role of CR on the health of the cardiovascular system, there are also some warnings in the scientific community regarding the indiscriminate application of CR regimen in humans. In fact, it has been reported in a 29 years old woman that a CR regimen can exacerbate the myocardial stress after high-intensity exercise [178].

## 5. Conclusions

Sirtuins are critical mediators of the beneficial effect of CR on signs of ageing and diseases. In this context, SIRT1 plays a major role compared to the other members of the sirtuin family. So far, the mechanisms underlying the mechanisms associated with detrimental effects of ageing are not fully elucidated. However, the possibility to affect some negative consequences of ageing in different species, including humans, is seriously entertained within the scientific community and nutritional interventions are some of the most studied strategies to promote a healthy life. In particular, experimental evidence indicates a positive role of CR in affecting the decay of the organismal performances with ageing; thus, we believe that CR can contribute to increase health span in many species, and most probably also in humans. To reach this goal CR should preserve the efficiency of skeletal and heart muscles, which is fundamental for the wellbeing of vertebrates in general, and humans in particular, and is compromised during ageing and in age-related diseases. However, CR might not be a strategy that is easily applicable by many, especially in the long run, because it is very demanding for many. Therefore, the present and future knowledge about the role of sirtuins in muscle tissues, especially under conditions of CR, might offer alternative strategies to dietary restrictions since many compounds that stimulate sirtuins and the same anti-ageing pathways induced by CR, are being identified and developed.

## Figures and Tables

**Table 1:** Sirtuin-mediated anti-ageing effects of Calorie Restriction in cardiac and skeletal muscle.

Skeletal Muscle	Cardiac Muscle
↑ Mass and function	Altered lipid metabolism
↑ Satellite cell activity	Hypertrophy
↓ Inflammation	↓ Inflammation
↑ Resistance to stress	↑ Resistance to stress
↑ Insulin sensitivity	↑ Insulin sensitivity
↑ Autophagy	↑ Autophagy
Mitochondrial dysfunction	Mitochondrial dysfunction
↓ DNA damage	

## Abbreviations

4E-BP1	eIF4E-binding protein1
AMPK	AMP-activated protein kinase
CR	Calorie restriction
eNOS	Endothelial nitric oxide synthase
FOXOs	Forkhead box O
GLUT-4	Glucose transporter type 4
HSC	Hematopoietic stem cell
I/R	Ischemia/reperfusion
IGF-1	Insulin growth factor type 1
KO	Knockout
MEF2	Myocyte enhancer factor-2
Mn-SOD	Manganese-dependent superoxide dismutase
mTOR	Mammalian target of rapamycin
MYOD	Myogenic differentiation factor
NAMPT	Nicotinamide phosphoribosyltransferase
NF-κB	Nuclear factor kappa-light-chain-enhancer of activated B cells
PDK	Pyruvate dehydrogenase kinase
PEPCK1	Phosphoenolpyruvate carboxykinase 1
PGC-1α	Peroxisome proliferator-activated receptor gamma coactivator 1-alpha
PPAR	Peroxisome proliferator-activated receptor
ROS	Reactive oxygen species
S6K1	P70S6 kinase 1
SIRT	Sirtuin
SREBP-1C	Sterol regulatory element-binding protein 1
